# ANP32e Binds Histone H2A.Z in a Cell Cycle-Dependent Manner and Regulates Its Protein Stability in the Cytoplasm

**DOI:** 10.1080/10985549.2024.2319731

**Published:** 2024-03-14

**Authors:** Yasmin Dijkwel, Gene Hart-Smith, Sebastian Kurscheid, David J. Tremethick

**Affiliations:** a The John Curtin School of Medical Research, The Australian National University, Canberra, Australia; b School of Biotechnology and Biomolecular Sciences, University of New South Wales, Sydney, Australia; c Australian Proteome Analysis Facility, Macquarie University, Sydney, Australia

**Keywords:** ANP32e, histone, histone chaperone, H2A.Z, protein stability

## Abstract

ANP32e, a chaperone of H2A.Z, is receiving increasing attention because of its association with cancer growth and progression. An unanswered question is whether ANP32e regulates H2A.Z dynamics during the cell cycle; this could have clear implications for the proliferation of cancer cells. We confirmed that ANP32e regulates the growth of human U2OS cancer cells and preferentially interacts with H2A.Z during the G1 phase of the cell cycle. Unexpectedly, ANP32e does not mediate the removal of H2A.Z from chromatin, is not a stable component of the p400 remodeling complex and is not strongly associated with chromatin. Instead, most ANP32e is in the cytoplasm. Here, ANP32e preferentially interacts with H2A.Z in the G1 phase in response to an increase in H2A.Z protein abundance and regulates its protein stability. This G1-specific interaction was also observed in the nucleoplasm but was unrelated to any change in H2A.Z abundance. These results challenge the idea that ANP32e regulates the abundance of H2A.Z in chromatin as part of a chromatin remodeling complex. We propose that ANP32e is a molecular chaperone that maintains the soluble pool of H2A.Z by regulating its protein stability and acting as a buffer in response to cell cycle-dependent changes in H2A.Z abundance.

## Introduction

Upregulation of ANP32e expression is associated with increased proliferation of cancers including triple-negative breast cancer,^1^ childhood acute myeloid leukemia,^2^ mesothelioma,^3^ thyroid carcinoma,^4^ medulloblastoma,^5^ pancreatic cancer,^2^ and lung cancer.^6^ This upregulation is associated with poor prognostic outcomes for cancer patients.^1–3^^,^^5–7^ The evidence suggesting that ANP32e promotes cancer cell proliferation also suggests that it may play a role in regulating cell proliferation and cell cycle progression.

ANP32e is part of the ANP32 family of proteins that have protein phosphatase 2 (PPP2)-inhibitor functions. ANP32 family members also have diverse other roles including regulation of chromatin modification, modulation of apoptotic caspase, and facilitation of intracellular protein transport.^8^ ANP32e was first identified as an inhibitor of PP2A in the early 2000s^9^^,^^10^ but was more recently described as a chaperone for the histone H2A variant H2A.Z.^11^ Studies have suggested that ANP32e is involved in the removal of H2A.Z from chromatin as part of the p400 and INO80 chromatin remodeling complexes,^12^ stabilization of H2A.Z protein by preventing its proteasome-mediated degradation,^13^ and the nuclear import of H2A.Z.^13^ Most data agree that ANP32e regulates the abundance of H2A.Z in chromatin because depletion of ANP32e from cells leads to an increase in H2A.Z abundance in chromatin.^12^^,^^14–17^ However, the exact mechanism of action of ANP32e is unclear. For example, although ANP32e depletion increases H2A.Z binding to enhancers and promoters,^12^^,^^14^^,^^15^ ANP32e is incapable of binding nucleosomes but is instead involved in resolving H2A.Z–H2B–DNA aggregates.^14^

It has been reported that ANP32e associates with and functions as part of p400 and INO80 chromatin remodeling complexes,^12^^,^^18^ although this has also been disputed.^19^^,^^20^ ANP32e has been suggested to be involved in mediating the nuclear import and protein stability of H2A.Z.^13^ There are discrepancies in reports about the cellular localization of ANP32e—whether it is cytoplasmic, nuclear, or both.^9^^,^^13^^,^^19^^,^^21^ These diverse results suggest that ANP32e plays multiple, potentially context-dependent, roles.

H2A.Z is a variant of the histone H2A. Given that it localizes to transcription start sites (TSSs) of active and inactive genes,^22^ the main research focus is on its role in gene expression. However, H2A.Z also localizes to other genomic regions such as heterochromatic regions, including centromeres and telomeres,^23–29^ and its localization in these regions is important for the survival of cells, especially during development.^26^,^30^ In addition, the deposition of H2A.Z at specific genomic regions has been shown to be cell cycle regulated. During the cell cycle, there is a redistribution of H2A.Z around the TSS, a global reduction in H2A.Z abundance at the TSS, and a shift from homo- to heterotypic nucleosomes in S-G2-M phase, which coincide with an increase in H2A.Z abundance at centromeres.^29^ However, the mechanism regulating this redistribution is unknown. H2A.Z is also implicated in cancer formation and progression, and upregulation of H2A.Z is associated with increased cancer cell proliferation and poor prognosis for cancer patients via different mechanisms.^31–35^

ANP32e and H2A.Z are thought to play important roles in cell proliferation, cell cycle progression, and cancer. However, a link between the two proteins in cell cycle progression has not been established, and we performed this study to examine this link further. We found that ANP32e depletion led to a decrease in cell proliferation and mitotic cell population, findings that implicate ANP32e as a potential oncogene in the U2OS cell line. To identify potential roles of ANP32e in the cell, we used an unbiased MS approach to investigate whether the protein interactome of ANP32e changes during the cell cycle. We identified a preferential interaction between ANP32e and H2A.Z during G1 phase compared with G2-M phase of the cell cycle in the nucleoplasm. We also found that ANP32e did not mediate the removal of H2A.Z from chromatin, was not a stable component of any remodeling complex, and was not strongly associated with chromatin. Most ANP32e was found not in the nucleus, but in the cytoplasm in U2OS cells. In the cytoplasm, this ANP32e–H2A.Z interaction was greater in G1 compared with G2-M phase of the cell cycle. However, unlike the nucleoplasm, H2A.Z abundance in the cytoplasm in G1 was double that of G2-M. Finally, we found that depletion of ANP32e resulted in an almost complete loss of H2A.Z protein in the cytoplasm but not in the nucleoplasm. Using SNAP-tagged H2A.Z, we found that ANP32e depletion caused a decrease in the abundance of nascent H2A.Z protein in both the cytoplasm and nucleus. Taken together, these results suggest that ANP32e is a molecular chaperone for H2A.Z that prevents its degradation in the cytoplasm, thereby ensuring that the required amounts of H2A.Z are imported into the nucleus.

## Results

### ANP32e plays a role in regulating cell growth in the U2OS cancer cell line

ANP32e is considered to be a potential oncogene and is thought to regulate cell proliferation.^1–7^^,^^36^ In this study, we investigated whether ANP32e plays a role in regulating cell proliferation in the U2OS human osteosarcoma cell line. This cell line was chosen as the model because it expresses WT p53 and has intact cell cycle checkpoints,^37^ which are features of a “normal” cell cycle compared with other commonly used cancer cell lines. Further, as described below, ANP32e depletion in U2OS cells caused a reduction in cell growth, which suggests that ANP32e plays an important role in regulating the cell cycle in these cells.

**Figure 1.**
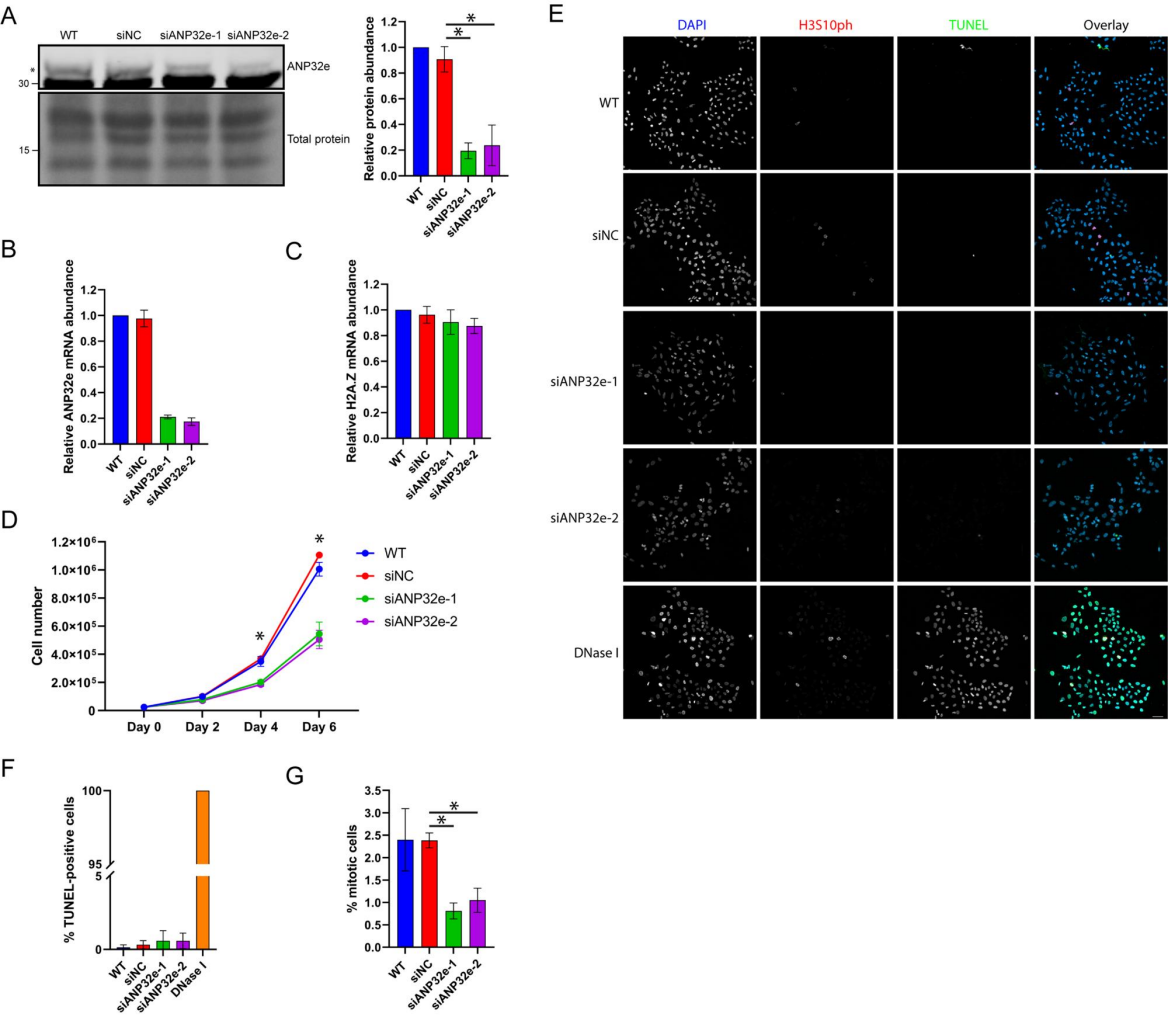
ANP32e regulates cell proliferation of U2OS cells. (A) Protein levels of ANP32e were measured by Western blotting after 72 h incubation with no siRNA (WT), negative control siRNA (siNC), or one of two independent siRNAs targeting ANP32e (siANP32e-1 or siANP32e-2). Quantification is shown in the right panel (n = 3). Error bars represent SD. *p < 0.05. (B) mRNA levels of ANP32e and (C) H2A.Z were measured by RT-qPCR after 72 h incubation with siRNA (n = 3). Error bars represent SD. *p < 0.05. (D) Following siRNA treatment, cell proliferation was determined by counting total cell numbers on days 0, 2, 4, and 6 post-siRNA treatment (n = 3). *p < 0.05. (E) Confocal images of siRNA-treated, asynchronously growing cells stained with Phospho-Histone H3 (Ser10) Antibody, TUNEL, and DAPI. A representative image of each sample is shown. Scale bar = 50 µm. (F) Quantification of apoptotic cells as determined by TUNEL assay and DAPI staining followed by confocal imaging. DNase I-treated cells were used as a positive control (n = 3). Error bars represent SD. *p < 0.05. (G) Quantification of confocal images taken of siRNA-treated, asynchronously growing cells stained with Phospho-Histone H3 (Ser10) Antibody and DAPI. The total numbers of cells and mitotic cells were counted at 10× magnification in five fields of views per experiment (n = 3). Error bars represent SD. *p < 0.05.

U2OS cells were depleted of ANP32e using two independent siRNAs (siANP32e-1 and siANP32e-2). An siRNA comprising a random nontargeting sequence was used as a negative control and termed siNC. Protein levels of ANP32e were reduced by approximately 80% as analyzed by Western blotting (Figure 1A), and mRNA levels were also reduced by approximately 80% as analyzed by RT-qPCR (Figure 1B). H2A.Z mRNA levels were not affected upon ANP32e depletion (Figure 1C).

To identify a potential role of ANP32e in the cell cycle we examined cell growth, cell death, and mitotic cell number. ANP32e depletion significantly decreased cell growth rate (Figure 1D). There was no increase in cell death as determined by TUNEL staining (Figure 1E and F). Interestingly, the proportion of cells in mitosis decreased in the ANP32e-depleted cell population, as visualized by H3S10ph staining and confocal imaging (Figure 1E and G). In WT and siNC cultures, approximately 2.5% of cells were in mitosis, but only 1% of siANP32e cells were in mitosis. These results suggest that ANP32e is required for cell proliferation.

### ANP32e preferentially interacts with H2A.Z during G1 phase in the nucleoplasm

**Figure 2.**
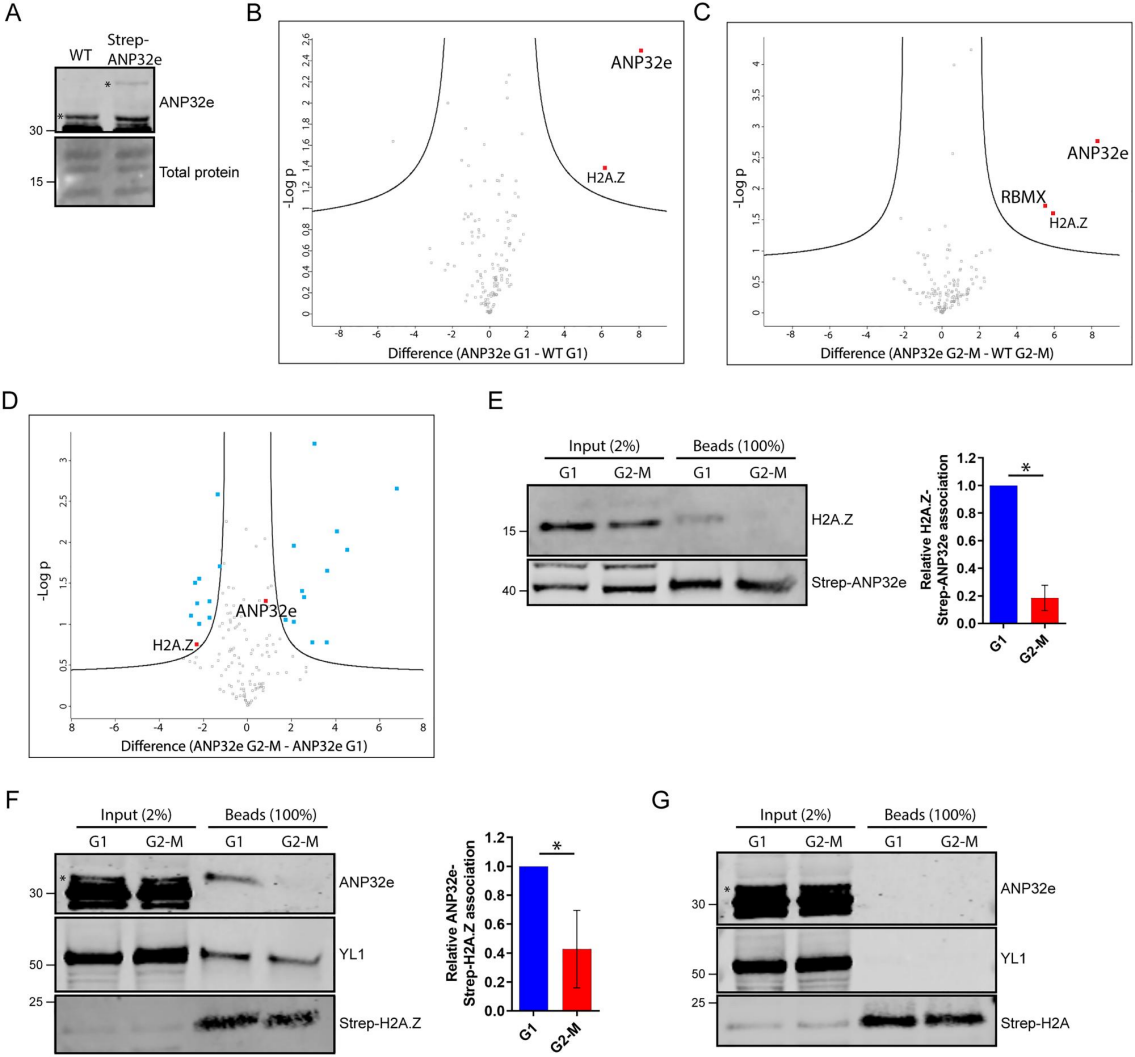
ANP32e preferentially interacts with H2A.Z during G1 phase of the cell cycle. (A) Western blot analysis of whole-cell lysates of cells not expressing Strep-tagged protein (WT) and cells expressing Strep–ANP32e identified with antibody against endogenous protein. The Pierce reversible total protein stain was used as a loading control. The top asterisk indicates Strep–ANP32e protein, and the bottom asterisk indicates endogenous ANP32e protein. (B) Volcano plot showing putative nucleoplasmic Strep–ANP32e interactants in G1 phase of the cell cycle, or (C) G2-M phase of the cell cycle. Statistical analysis was carried out with Perseus software. Proteins are shown in red dots if *p* < 0.05 and log2-fold difference > 1. (D) Volcano plot showing differential protein abundance (*p* < 0.05 and log2-fold difference > 1 or < −1) in Strep–ANP32e pulldowns in G1 phase (left) or G2-M phase (right) phase of the cell cycle. Proteins identified as putative Strep–ANP32e interactants in (B) and (C) are shown in red dots. Proteins identified as non-specific background in (B) and (C) but that did show differential protein abundance are shown in blue dots. (E) Strep–ANP32e was affinity purified from nuclear extract of cells synchronized in G1 or G2-M phase. The abundance of associated H2A.Z was measured by Western blotting. The abundance of H2A.Z and ANP32e protein was normalized to that of the total Strep-tagged protein. The right panel shows quantification of replicates (n = 3). Error bar represents SD. *p < 0.05. (F) As in (E), except Strep–H2A.Z was affinity purified, and the association of endogenous ANP32e and YL1 was measured (n = 3). Error bar represents SD. *p < 0.05. (G). As in (E), except Strep–H2A was affinity purified as a negative control. No ANP32e or YL1 was detected.

Next, we aimed to identify whether the cell cycle role of ANP32e is related to H2A.Z. We used an affinity purification mass spectrometry (AP-MS) approach to identify potential changes in the ANP32e interactome in G1 and G2-M phases of the cell cycle. U2OS cells stably expressing Strep–ANP32e were produced, and the expression levels of endogenous versus tagged ANP32e were measured. Approximately 10% of total ANP32e protein was Strep-tagged (Figure 2A). Cells were synchronized in G1 or G2-M phase of the cell cycle (Supplementary material, Figure S1), and AP of Strep–ANP32e was performed from nucleoplasmic subcellular fractions. This was followed by liquid chromatography-tandem mass spectrometry (LC-MS/MS) to identify the ANP32e–protein interactants using WT cells as a control for nonspecific background.

Proteins found to be present in Strep–ANP32e pulldown samples and absent in controls, or of significantly higher abundance in pulldown samples relative to controls (*p* < 0.05 and log2-fold difference > 1), were identified as putative ANP32e interactants and are shown in red dots in Figure 2B and C for G1 phase and G2-M phase, respectively. Changes in the abundance of proteins associated with ANP32e in G1 and G2-M phase were then determined. Proteins found to be of significantly different abundance in G1 relative to G2-M samples (*p* < 0.05 and log2-fold difference > 1 or < −1), but that were not putative ANP32e interactants are visualized in the volcano plot shown in Figure 2D in blue dots. The dots corresponding to H2A.Z (the only protein identified as an ANP32e interactant in both G1 and G2-M) and ANP32e are red. Proteins found not to significantly differ in abundance in G1 relative to G2-M are shown as black dots. All putative interactants are listed in Table S1 (Supplementary material).

Interestingly, the MS data suggested a greater association between H2A.Z and ANP32e in G1 compared with G2-M phase in the nucleoplasm. This was validated by AP followed by Western blotting. Both the association of endogenous ANP32e with Strep–H2A.Z and Strep–ANP32e with endogenous H2A.Z were analyzed to exclude the possibility that this interaction is dependent on the presence of the Strep-tag. Strep–H2A pulldowns were used as negative controls. Consistent with the MS data, we observed a greater association between ANP32e and H2A.Z in Strep–ANP32e pulldowns (Figure 2E) as well as in Strep–H2A.Z pulldowns (Figure 2F) in G1 compared with G2-M phase. This was specific for ANP32e, because the other known H2A.Z chaperone YL1 was pulled down equally by Strep–H2A.Z in G1 and G2-M (Figure 2F). ANP32e was not an interactant of Strep–H2A in either cell cycle fraction (Figure 2G). These findings demonstrate that, in the nucleoplasm, ANP32e preferentially associates with H2A.Z during G1 phase of the cell cycle.

Previous studies have suggested that ANP32e associates with the p400^12^ and INO80^18^ complexes. However, it has also been suggested that ANP32e functions independently of the p400 complex during repair of double-strand breaks.^19^ In this analysis, no components of the p400 or INO80 complex were identified as ANP32e interactants in the nucleoplasm. This suggests that ANP32e may not be a stable component of p400 or INO80 in the U2OS cell line.

Taken together, these findings suggest that ANP32e interacts with H2A.Z in the nucleoplasm in a cell cycle-dependent manner. However, the reason for this is unknown. Previous studies have suggested that ANP32e is involved in the removal of H2A.Z from chromatin,^12^^,^^14^^,^^15^^,^^19^^,^^21^ stabilization of H2A.Z protein,^13^ and/or nuclear import of H2A.Z.^13^ We investigated these possibilities in further experiments.

### ANP32e does not play a role in removing H2A.Z from chromatin

**Figure 3.**
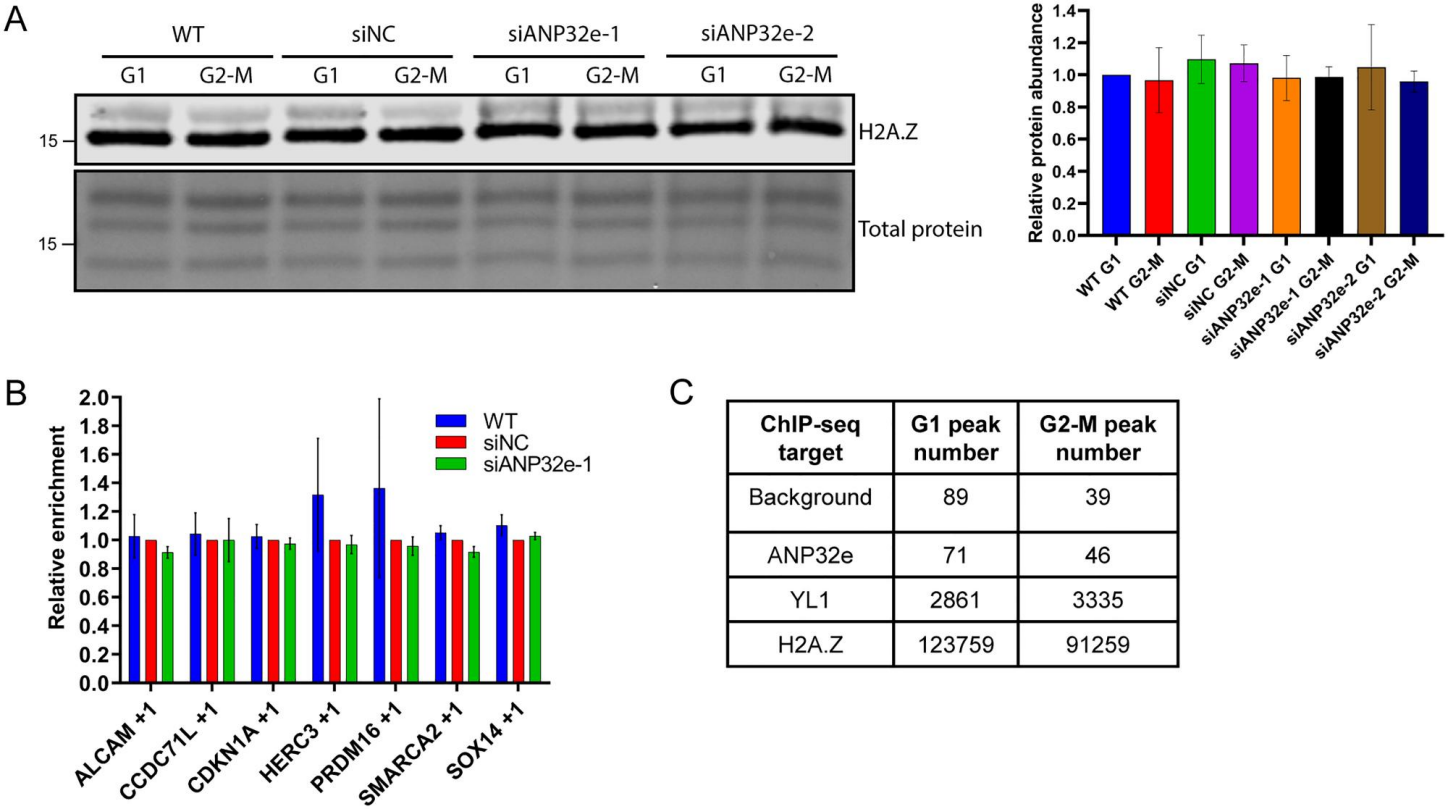
ANP32e does not remove H2A.Z from chromatin. (A) Cells were treated for 72 h with no siRNA (WT), negative control siRNA (siNC), or siRNAs targeting ANP32e (siANP32e-1 or siANP32e-2), and then synchronized in G1 or G2-M phase. Cells were fractionated, and the chromatin pellet fraction was analyzed by Western blotting to measure H2A.Z abundance. The Pierce total protein stain was used as a loading control. Quantification of H2A.Z signal normalized to total protein is shown on the right (n = 3). Error bars represent SD. (B) H2A.Z abundance at +1 nucleosomes at seven gene promoters was determined by ChIP-qPCR after siRNA treatment for 72 h (n = 3). Error bars represent SD. (C) Total numbers of peaks identified in ChIP-sequencing experiments of negative control (WT), Strep–ANP32e-associated DNA, Strep–YL1-associated DNA, or Strep–H2A.Z-associated DNA in cells synchronized in G1 or G2-M phase. Peak numbers indicate after the normalization to input.

To identify whether the G1-specific role of ANP32e is to remodel H2A.Z-containing nucleosomes, we first investigated whether ANP32e affects the level of H2A.Z chromatin incorporation. After ANP32e depletion, cells were synchronized in G1 or G2-M phase of the cell cycle, subcellular fractionation was performed, and the chromatin pellet was analyzed for the abundance of H2A.Z. Upon depletion of ANP32e, the amount of H2A.Z associated with the chromatin pellet did not differ between G1 and G2-M phase (Figure 3A). This suggests that ANP32e is not involved in regulating the global association of H2A.Z with chromatin in either of these phases of the cell cycle.

To investigate further whether ANP32e is involved in the removal of H2A.Z from chromatin, ANP32e was depleted from cells and ChIP-quantitative PCR (qPCR) analysis was performed to measure the abundance of H2A.Z at seven promoter +1 nucleosomes known to be bound by H2A.Z (Supplementary material, Figure S2). Asynchronous cells were used because most asynchronous cells are in G1 phase (Supplementary material, Figure S4). Upon ANP32e depletion, no difference in H2A.Z abundance at any of the promoter +1 nucleosomes was seen (Figure 3B). This finding suggests that ANP32e does not play a role in regulating the incorporation of H2A.Z at the seven promoters investigated in the U2OS cell line.

Because ANP32e does not seem to play a major role in regulating H2A.Z chromatin incorporation, we questioned to what extent ANP32e is associated with chromatin. We performed a ChIP-sequencing experiment with Strep–ANP32e. WT U2OS cells were used as a negative control. Cells expressing Strep–YL1 were used as a positive control, because YL1 is known to bind chromatin as part of the p400 and SRCAP complexes.^20^^,^^38^ Cells expressing Strep–H2A.Z were used as a comparison. For the negative control WT cells, 89 and 39 peaks were identified in G1 and G2-M phase, respectively. For ANP32e, 71 and 46 peaks were identified in G1 and G2-M, respectively. In contrast, for YL1, 2861 and 3335 peaks were identified in G1 and G2-M, respectively. H2A.Z had 123,759 and 91,259 peaks identified, respectively (Figure 3C). H2A.Z was located throughout the genome, and YL1 was enriched at promoters, as expected (Supplementary material, Figure S3). The peak numbers indicate that ANP32e does not associate strongly with chromatin and suggest further that the G1-specific role of ANP32e is not related to H2A.Z nucleosome exchange. These data suggest that ANP32e does not remove H2A.Z from chromatin directly but plays a different role in U2OS cells.

### ANP32e is present in the cytoplasm where it interacts with H2A.Z in an abundance-dependent manner

**Figure 4.**
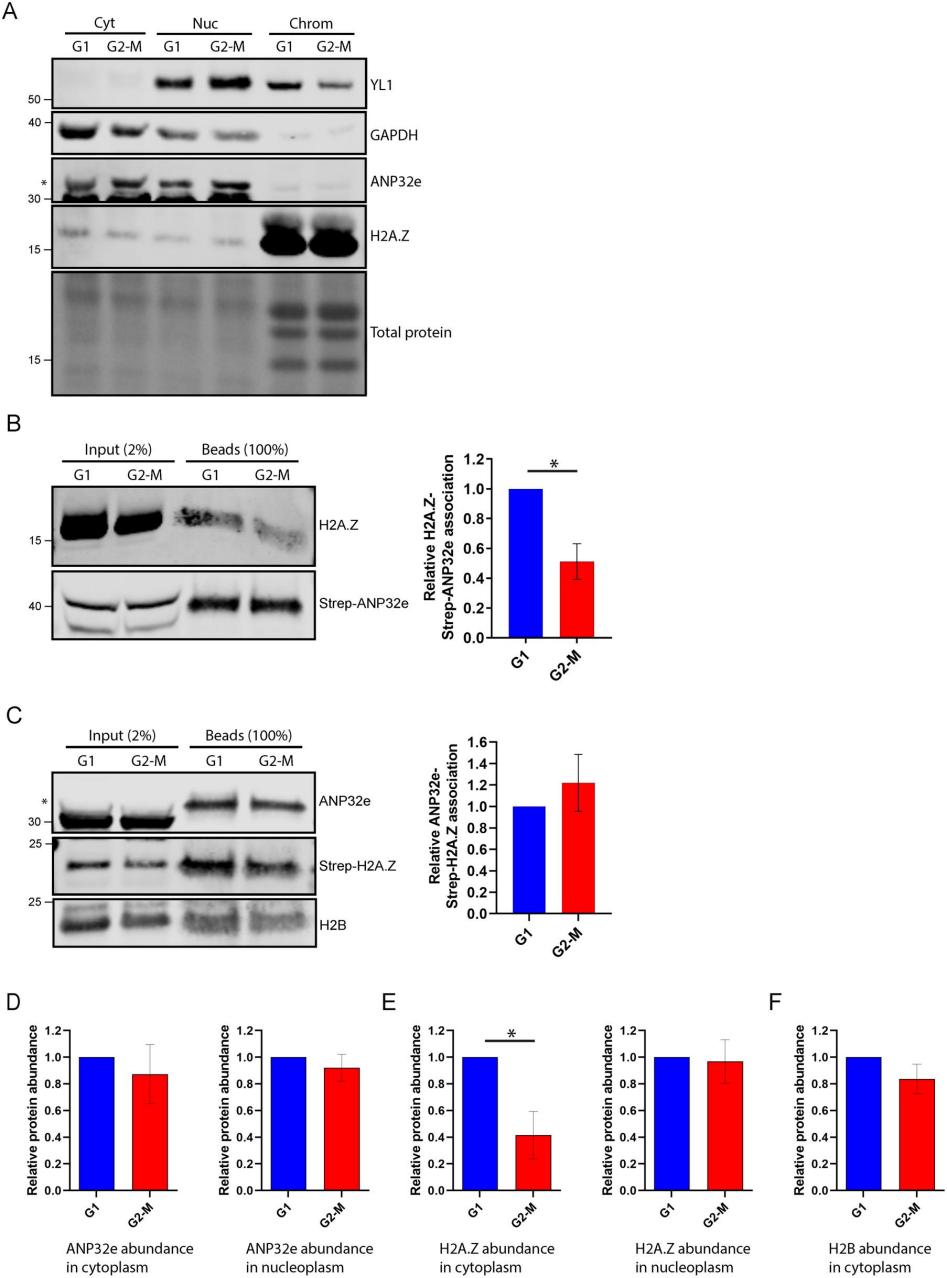
ANP32e interacts with H2A.Z in the cytoplasm in proportion to H2A.Z protein abundance. (A) Equal proportions of cytosolic (Cyt), nuclear (Nuc), and chromatin pellet (Chrom) fractions from cells synchronized in G1 or G2-M phase were analyzed by Western blotting to measure the abundance of YL1, GAPDH, ANP32e, and H2A.Z. The asterisk indicates the ANP32e band. (B) Strep–ANP32e was affinity purified from cytoplasmic extract of cells synchronized in G1 or G2-M phase. The abundance of associated H2A.Z was measured by Western blotting and normalized to that of the total Strep-tagged protein. The right panel shows quantification of replicates (n = 3). Error bar represents SD. *p < 0.05. (C) As in (B), except Strep–H2A.Z was affinity purified and the association of endogenous ANP32e and H2B was measured. The right panel shows the quantification (n = 3). Error bar represents SD. (D) The total abundance of ANP32e protein in the cytoplasm (left) and nucleoplasm (right) of cells synchronized in G1 or G2-M phase was quantified by Western blotting (shown in (A)) and normalized to total protein (n = 4). Error bar represents SD. (E) The total abundance of H2A.Z protein in the cytoplasm (left) and nucleoplasm (right) of cells synchronized in G1 or G2-M phase was quantified by Western blotting (shown in (A)) and normalized to total protein (n = 3). Error bar represents SD. *p < 0.05. (F) The total abundance of H2B protein in the cytoplasm of cells synchronized in G1 or G2-M phase was quantified by Western blotting (shown in (C)) and normalized to total protein (n = 4). Error bar represents SD.

Previous studies have suggested that ANP32e can localize to the nucleus or the cytoplasm.^9^^,^^13^^,^^19^^,^^21^ Given that the data presented here suggest that ANP32e does not play a major role in H2A.Z chromatin exchange in the nucleus, we examined the subcellular localization of ANP32e. Cells were synchronized in G1 or G2-M phase of the cell cycle and then subjected to subcellular fractionation into cytoplasmic, nucleoplasmic, and chromatin pellet fractions. Western blotting was used to analyze the abundance of ANP32e in equal proportions of these fractions. We observed that most ANP32e localized to the cytoplasm (Figure 4A).

Next, we examined whether ANP32e interacts with H2A.Z in the cytoplasm. Indeed, Strep–ANP32e interacted with H2A.Z in the cytoplasm (Figure 4B), and this interaction was greater in G1 phase compared with G2-M phase (Figure 4B) (this was also observed in the nucleoplasm (Figure 2E)). However, when using Strep–H2A.Z, an equal amount of ANP32e was associated with Strep–H2A.Z in G1 and G2-M (Figure 4C). Interestingly, the abundance of ANP32e did not differ between G1 and G2-M in the cytoplasm or nucleoplasm (Figure 4D). However, the abundance of H2A.Z protein in the cytoplasm, but not the nucleoplasm, was greater in G1 compared with G2-M (Figure 4E). This pattern was not observed for the canonical histone H2B (Figure 4F). Thus, the greater interaction between ANP32e and H2A.Z in the cytoplasm in G1 phase is correlated with the total abundance of H2A.Z protein.

### ANP32e regulates H2A.Z protein stability in the cytoplasm

**Figure 5.**
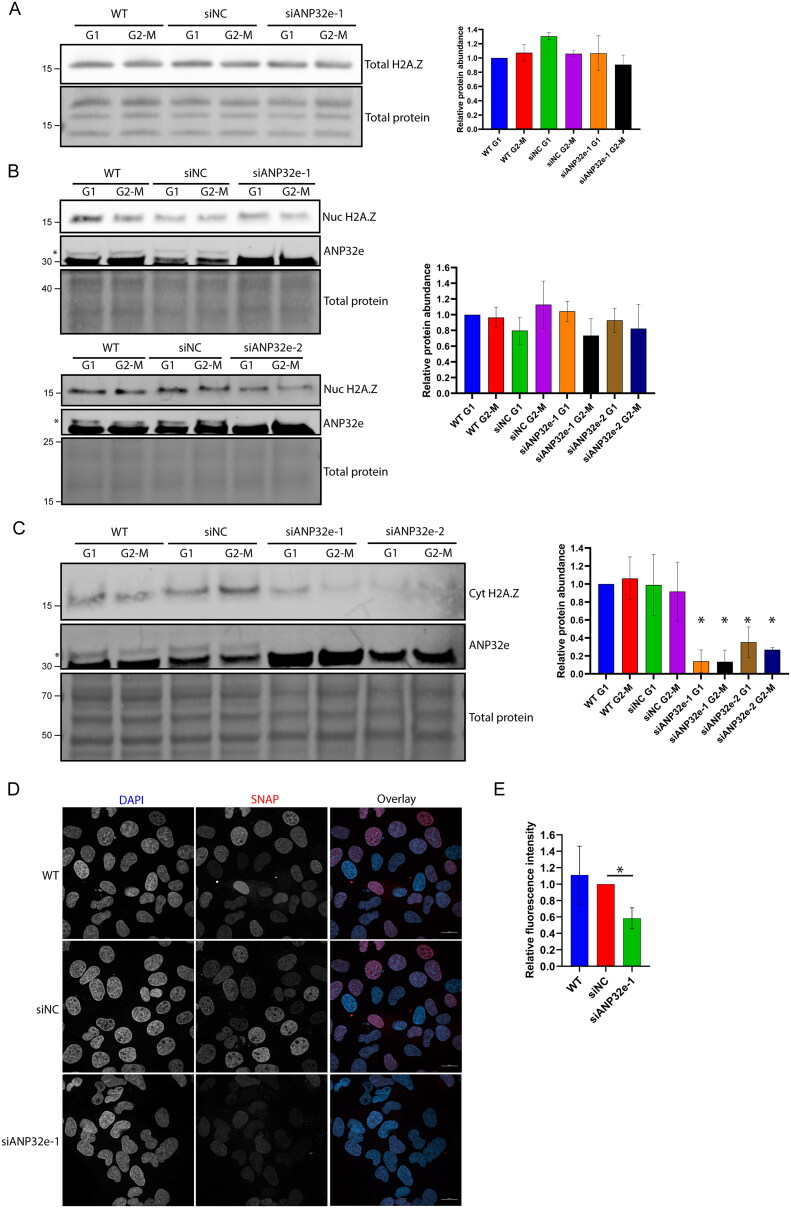
ANP32e regulates H2A.Z protein stability in the cytoplasm. (A) Cells were treated for 72 h with no siRNA (WT), negative control siRNA (siNC), or siRNA targeting ANP32e (siANP32e-1), and then synchronized in G1 or G2-M phase. Whole-cell lysates were analyzed by Western blotting for total H2A.Z protein. The Pierce total protein stain was used as a loading control. Quantification of H2A.Z signal normalized to total protein is shown on the right (n = 3). Error bars represent SD. (B) Cells were treated for 72 h with siRNA and synchronized in G1 or G2-M phase. Cells were fractionated and the nucleoplasmic fraction was analyzed by Western blotting to measure H2A.Z and ANP32e abundance. The Pierce total protein stain was used as a loading control. Quantification of H2A.Z signal normalized to total protein is shown on the right (n = 3). Error bars represent SD. (C) Cells were treated for 72 h with siRNA and synchronized in G1 or G2-M phase. Cells were fractionated, and the cytoplasmic fraction was analyzed by Western blotting for H2A.Z and ANP32e abundance. The Pierce total protein stain was used as a loading control. Quantification of H2A.Z signal normalized to total protein is shown on the right (n = 3). Error bars represent SD. *p < 0.05. (D) Cells expressing SNAP–H2A.Z were blocked treated for 48 h with siRNA. The SNAP-tag was blocked with SNAP-Cell Block to prevent fluorescence, and nascent SNAP–H2A.Z protein was produced for 48 h. The SNAP-tag was labeled with SNAP-Cell 647-SiR and imaged using confocal microscopy. DAPI was used to visualize nuclei. A representative image of each sample is shown. Scale bar = 20 µm. (E) Quantification of nascent nuclear SNAP–H2A.Z using images from (D). SNAP-tag fluorescence was quantified only in regions that were stained with DAPI (n = 3). Error bars represent SD. *p < 0.05.

Thus far, our results are consistent with those of a previous study showing that ANP32e is involved in regulating H2A.Z protein stability in the cytoplasm and in the nuclear import of H2A.Z.^13^ To determine whether ANP32e is required for maintaining proper H2A.Z protein levels in U2OS cells, Western blotting of total cell lysates of synchronized cells was used to measure the abundance of endogenous H2A.Z. Total H2A.Z protein levels did not change significantly after ANP32e depletion (Figure 5A), which suggests ANP32e does not play a major role in regulating the stability of total cellular H2A.Z. This is not surprising given that most H2A.Z is contained within chromatin, whereas the bulk of ANP32e is in the cytoplasm.

To investigate the role of ANP32e in regulating H2A.Z protein stability in the cytoplasm, ANP32e was depleted using siRNA, cells were synchronized in G1 or G2-M phase of the cell cycle, and subcellular fractionation was performed. The abundance of H2A.Z in the nucleoplasmic fraction was analyzed by Western blotting. After ANP32e depletion, there was no difference in H2A.Z protein abundance in the nucleoplasm between G1 and G2-M phase (Figure 5B). This finding suggests that the role of ANP32e is not to maintain the stability of H2A.Z in the nucleoplasm.

Next, the cytoplasmic fraction was analyzed in the same manner. After depletion of ANP32e, H2A.Z protein was significantly reduced in the cytoplasm in both the G1 and G2-M phase fractions (Figure 5C). This finding suggests that ANP32e is involved in maintaining H2A.Z protein stability in the cytoplasm. However, it is also possible that ANP32e prevents the nuclear import of H2A.Z and, thus, depletion of ANP32e may upregulate the nuclear import and decrease the cytoplasmic abundance of H2A.Z. To examine whether ANP32e regulates H2A.Z protein stability, we used U2OS cells expressing SNAP-tagged H2A.Z to investigate the effect of ANP32e depletion on nascent H2A.Z protein. SNAP–H2A.Z was blocked 48 h after the depletion of ANP32e by siRNA, after which nascent SNAP–H2A.Z was allowed to be produced for a further 48 h. Confocal imaging and ImageJ analysis were performed to quantify the amount of SNAP–H2A.Z within the nuclei, which were identified using DAPI staining. Representative images of each of the controls and ANP32e-depleted cells are shown in Figure 5D. Interestingly, upon the depletion of ANP32e, the amount of SNAP–H2A.Z in the nucleus was reduced to about 60% of that observed in the siNC control (Figure 5E). These results further support the conclusion that ANP32e is required for maintaining H2A.Z protein stability and that loss of ANP32e reduces the amount of H2A.Z imported into the nucleus.

## Discussion

The main findings of this study are as follows: 1) ANP32e plays a role in regulating cell growth in the U2OS cancer cell line; 2) ANP32e preferentially interacts with H2A.Z during G1 phase in the nucleoplasm and cytoplasm; 3) ANP32e does not regulate the level of H2A.Z incorporated into chromatin and is not part of an H2A.Z-dependent chromatin remodeling complex; 4) ANP32e is not bound to chromatin but instead is present mainly in the cytoplasm, where it associates with H2A.Z; and 5) ANP32e regulates H2A.Z protein stability in the cytoplasm, thereby indirectly affecting its import into the nucleus. These results challenge the idea that the major role of ANP32e is in chromatin remodeling by removing or depositing H2A.Z into chromatin. Rather, our data suggest that ANP32e is a molecular chaperone required for stabilization of H2A.Z in the cytoplasm following protein synthesis in a cancer context.

### ANP32e is not a chromatin remodeler but a molecular chaperone for H2A.Z

ANP32e is thought to be involved in the removal of H2A.Z from chromatin by being part of the p400^12^ and INO80^18^ complexes. However, we found here that, in the U2OS cell line, ANP32e was not involved in regulating the incorporation of H2A.Z into chromatin globally or specifically into the +1 promoter nucleosomes of several H2A.Z-enriched genes. Further, in this study, ANP32e was not strongly associated with chromatin and no interactions with components of the p400 and INO80 remodeling complexes were observed in AP-MS experiments. Other studies have also failed to identify ANP32e as part of these remodeling complexes or functioning as a remodeler of H2A.Z-containing chromatin. These findings suggest that ANP32e does not have a universal function in removing H2A.Z from chromatin.

Instead, ANP32e appears to bind to H2A.Z in the cytoplasm, where it is required to stabilize H2A.Z. Our data suggest that ANP32e regulates the availability of H2A.Z by acting as its chaperone. This is supported further by the observation that the genomic sites where ANP32e is located do not necessarily overlap with sites where H2A.Z abundance changes after depletion of ANP32e.^13^^,^^17^ Another study reported that co-depletion of ANP32e and H2A.Z increased H2A.Z content in chromatin despite a significant decrease in the overall protein levels of H2A.Z.^17^ Based on our results, this suggests that depletion of ANP32e could cause a shift from soluble to chromatin-bound H2A.Z through the function of ANP32e as a molecular chaperone. The exact mechanism by which ANP32e regulates H2A.Z protein stability in U2OS cells remains to be determined, however ANP32e may protect H2A.Z from proteasomal degradation through PP2A inhibition.^13^ Another possibility is that ANP32e functions to sequester H2A.Z in the cytoplasm in a cell cycle dependent manner similar to the 14-3-3 family of proteins, which interact with cell cycle regulatory proteins.^39^ It is also likely that ANP32e plays a direct role in the nuclear import of H2A.Z because it contains both a nuclear localization sequence and nuclear export signal,^40^^,^^41^ as suggested previously.^13^ Other ANP32 family members have also been shown to play a role in the nuclear import of histones.^8^ Further, the protein level of ANP32e does not fluctuate during the cell cycle, which suggests the constant adequate supply to buffer against changing levels of H2A.Z.

### Cell cycle-dependent interaction between ANP32e and H2A.Z

**Figure 6.**
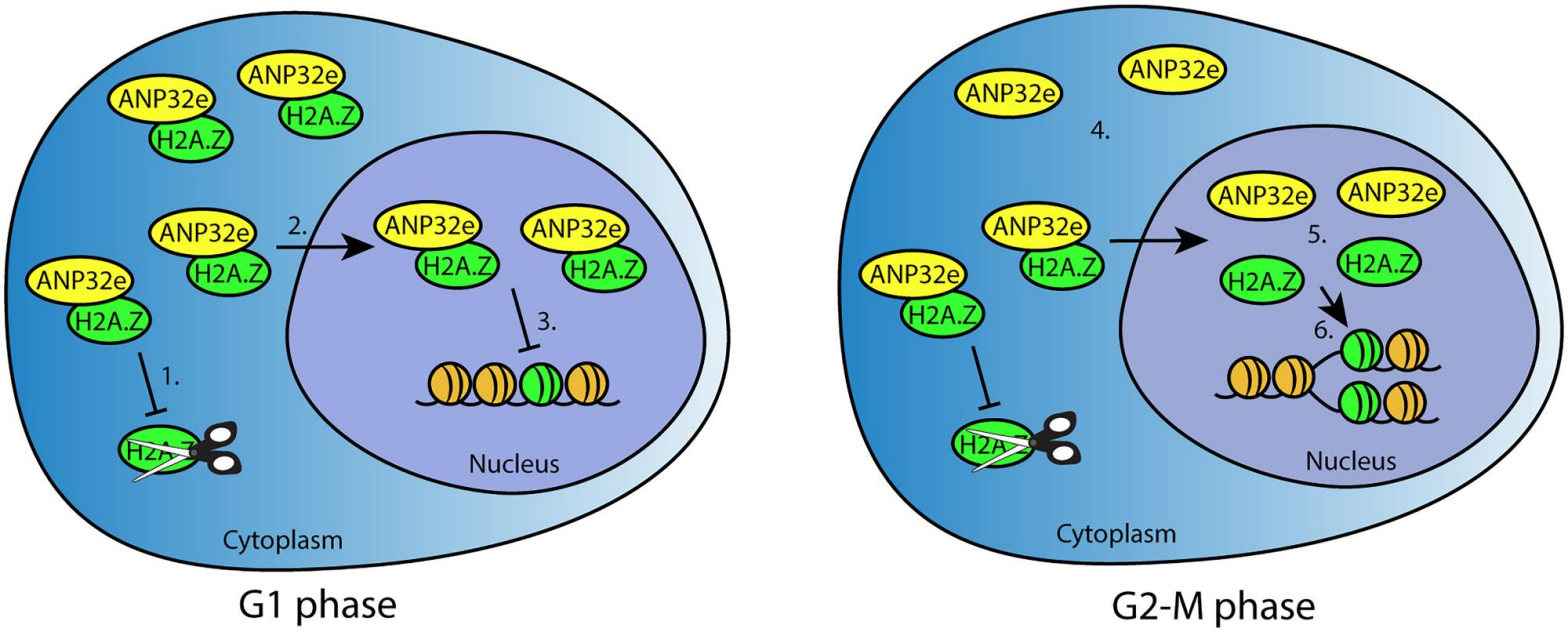
Model of ANP32e cell cycle functions related to H2A.Z. In G1 phase (left), ANP32e associates with H2A.Z in the cytoplasm and nucleoplasm. In the cytoplasm, ANP32e prevents the degradation of H2A.Z (1), which promotes its import into the nucleus (2). In the nucleus, ANP32e binding to H2A.Z prevents its incorporation into chromatin (3). In G2-M phase (right), the amount of H2A.Z protein associated with ANP32e in the cytoplasm is halved because of the decrease in the total abundance of H2A.Z protein (4). In the nucleus, the interaction between ANP32e and H2A.Z also decreases (5). However, this is not because of the decrease in the abundance of H2A.Z protein during G2-M. Release of H2A.Z from ANP32e allows the incorporation of H2A.Z into chromatin after DNA replication (6).

Although ANP32e associates with H2A.Z in the nucleoplasm,^12^^,^^14^ the data presented here show for the first time that this interaction increases in G1 phase of the cell cycle. Based on our data and previous publications, we speculate that, as a molecular chaperone, ANP32e has two cell cycle- and H2A.Z-related functions (Figure 6). First, given that H2A.Z is synthesized during G1 when its level is highest (U2OS cells spend most of their time in G1 (Supplementary material, Figure S4)), ANP32e binds to and stabilizes H2A.Z to ensure it is imported and available to be incorporated into chromatin throughout all stages of the cell cycle. ANP32e binds more H2A.Z in G1 phase of the cell cycle in both the cytoplasm and nucleoplasm. This is consistent with the nuclear import of H2A.Z–ANP32e from the cytoplasm during G1, when the H2A.Z protein level is at its highest. However, in the nucleoplasm, the G1-specific H2A.Z–ANP32e interaction is independent of the level of H2A.Z. We therefore speculate that a second cell cycle function of ANP32e occurs in the nucleoplasm where it acts to provide a store of H2A.Z to be released and incorporated into chromatin during DNA replication and the following G2-M phase of the cell cycle (Figure 6).

### ANP32e is required for cellular proliferation

We have shown here that ANP32e is required for proper cell proliferation in the U2OS cancer cell line. Upregulation of ANP32e expression is associated with increased proliferation of several cancers including triple-negative breast cancer,^1^ childhood acute myeloid leukemia,^2^ mesothelioma,^3^ thyroid carcinoma,^4^ medulloblastoma,^5^ pancreatic cancer,^7^ and lung cancer.^6^ Our data support these findings by showing that ANP32e is required for the growth of U2OS cells. However, we cannot exclude the possibility that ANP32e is required for normal cell growth given the importance of the proper regulation of H2A.Z dynamics. A comprehensive study of whether ANP32e is nuclear or cytoplasmic in malignant versus nonmalignant cells may be warranted to determine whether there is a link and whether this could explain the discrepancies in the function of ANP32e in different cellular contexts.

In conclusion, our findings suggest that there is a cell cycle-specific interaction between ANP32e and H2A.Z and that this interaction is important for the stability and import of H2A.Z into the nucleus. Future experiments are needed to understand better the primary function of ANP32e and whether its subcellular localization is regulated in a cell-type, developmental stage, or disease-specific manner.

## Materials and methods

### Cell culture

U2OS cells were maintained in Dulbecco’s modified Eagle’s medium (DMEM) (Sigma-Aldrich) containing 10% v/v fetal bovine serum, 1 mM sodium pyruvate, and 10 U/mL penicillin and streptomycin at 37 °C and in 5% CO_2_. Cells were confirmed as mycoplasma negative.

### Cell synchronization

Cells were synchronized at the G1–S phase boundary by adding 2 mM hydroxyurea (Sigma-Aldrich) to 70–80% confluent cells and incubated for 16 h. Cells were synchronized in metaphase by adding 50 ng/mL nocodazole (Sigma-Aldrich) to 70–80% confluent cells, and incubated for 18 h. Synchronization was confirmed by FACS.

### Establishment of stable overexpression cell lines

For N-terminal Twin-Strep-tag® tagging, ANP32e, H2A.Z, YL1, or H2A cDNA were cloned into pEXPR-IBA105 (IBA-Lifesciences). For N-terminal SNAP-tag® tagging, H2A.Z cDNA was cloned into pSNAP_f_ (New England BioLabs). U2OS cells were transfected with 2 µg of pEXPR-IBA105-ANP32e, -H2A.Z, -YL1, or -H2A, or pSNAP_f_-H2A.Z constructs using jetPRIME^®^ transfection reagent (Polyplus-transfection^®^ SA) according to the manufacturer’s protocol. Antibiotic selection was performed using complete growth medium containing 3 mg/mL G 418 (Sigma-Aldrich).

### siRNA transfection

U2OS cells were transfected using TriFECTa^®^ RNAi Kits (Integrated DNA Technologies) and Oligofectamine 2000 (Thermo Fisher Scientific) according to the manufacturer’s protocol.

### RNA extraction and RT-qPCR

RNA extraction was performed using a Qiagen RNeasy Mini kit as per the manufacturer’s protocol. cDNA synthesis was performed using 500 ng of RNA using a Qiagen QuantiTect Reverse Transcription kit as per the manufacturer’s protocol. A standard RT-qPCR reaction contained 1 µL of 5-fold-diluted cDNA, 5 µL of Power SYBR Green Master Mix (Thermo Fisher Scientific), and 2.5 µM of forward and reverse primers. Reactions were performed in triplicate in 384-well optical reaction plates (Applied Biosystems) on a QuantStudio 12K Flex Real-time PCR system (Applied Biosystems) at the Genome Discovery Unit—ACRF Biomolecular Resource Facility, The John Curtin School of Medical Research, Australian National University. To quantify the relative amounts of mRNA, the 2^–ΔΔCT^ method was used.^42^ The primers used for qPCR are listed in Table S2 (Supplementary material).

### TUNEL assay

The TUNEL assay was performed using the Click-iT™ Plus TUNEL Assay Kits for In Situ Apoptosis Detection (Invitrogen™) as per the manufacturer’s protocol. Cells were counterstained with DAPI. Apoptotic cells were detected using a Zeiss LSM800 with Airyscan and counted from at least five fields of view per experiment.

### Mitotic cell staining

Cells grown on coverslips were washed once with ice-cold PBS and fixed with 2% v/v formaldehyde in PBS for 15 min at room temperature. Cells were washed twice with PBS and incubated with permeabilization buffer (PBS supplemented with 0.25% v/v Triton-X-100) for 10 min at room temperature. Cells were incubated with blocking buffer (PBS containing 0.25% v/v Triton-X-100 and 3% w/v BSA) for 30 min at RT. Phospho-Histone H3 (Ser10) Antibody (New England BioLabs) was diluted in antibody dilution buffer (PBS containing 0.25% v/v Triton-X-100 and 1% w/v BSA) and incubated overnight at 4 °C. Coverslips were washed 3 × 5 min with PBS and incubated with secondary antibody diluted in antibody dilution buffer for 1 h at RT. Coverslips were washed three times with PBS. Cells were incubated with PBS containing 0.25% v/v Triton-X-100 and 1 µg/mL DAPI for 1 min at room temperature and washed once with PBS. Coverslips were mounted onto microscope slides with Vectashield mounting medium. Slides were imaged using a Zeiss LSM800 with Airyscan. Image quantification was performed using ImageJ.^43^

### SNAP-tag labeling

To block the SNAP-tag, cells grown on coverslips were incubated with medium supplemented with 10 µM SNAP-Cell^®^ Block (New England BioLabs) and incubated for 15 min in an incubator. SNAP-Cell^®^ Block was removed by washing cells twice with growth medium. Cells were incubated for 48 h to produce nascent protein. To label nascent protein, cells were incubated with growth medium supplemented with 3 µM SNAP-Cell^®^ 647-SiR (New England BioLabs) for 30 min in an incubator. Cells were washed three times with complete medium and incubated in fresh medium for 30 min, and the medium was replaced once more to remove unreacted substrate. Coverslips were processed and imaged using a Zeiss LSM800 with Airyscan.

### Western blot analysis

Proteins were separated by electrophoresis on Bolt® Plus Tris 4–12% gels (Thermo Fisher Scientific) using 1× Bolt^®^ MES SDS running buffer. Proteins were transferred to a nitrocellulose membrane using standard semi-dry blotting procedures with Bjerrum Schafer-Nielsen transfer buffer with SDS (48 mM Tris-HCl pH 9.2, 39 mM glycine, 0.03% w/v SDS, 15% v/v ethanol). Transferred proteins were visualized using a Pierce™ Reversible Protein Stain Kit (Thermo Fisher Scientific). The membrane was blocked using 5% w/v skim milk powder in TBST (20 mM Tris-HCl pH 7.5, 150 mM NaCl, 0.05% v/v Tween-20) for 1 h at room temperature. The membrane was incubated with primary antibody overnight at 4 °C, washed with TBST for a total of 30 min, incubated with secondary antibody for 1 h at room temperature, and then washed with TBST for another 30 min. Blots were imaged using an Odyssey CLx (LI-COR Biosciences) or an Amersham 680 Imager (GE Healthcare) using Immobilon Western Chemiluminescent HRP Substrate, at the Genome Discovery Unit—ACRF Biomolecular Resource Facility, The John Curtin School of Medical Research, Australian National University. Band intensity was quantified using ImageJ^36^ or ImageStudio (LI-COR Biosciences) software. The following antibodies were used for Western blotting: anti-H2A.Z (raised in our laboratory), anti-H2A.Z (Active Motif, 39113), anti-Strep-II (Abcam, ab76949), anti-YL1 (Abcam, ab112055), anti-ANP32e (Abcam ab5993), and anti-H2B (Abcam, ab1790).

### Subcellular fractionation

Cells were detached from culture vessels with PBS containing 125 mM EDTA and pelleted by centrifugation at 300 × g for 3 min. The cell pellet was washed once with ice-cold PBS, and the volume of the cell pellet approximated by eye. Five volumes of Buffer E containing protease, phosphatase, and histone deacetylase inhibitors (20 mM HEPES, 10 mM potassium chloride, 1.5 mM MgCl_2_, 1 mM DTT, 1× Roche EDTA-free protease inhibitor cocktail, 1× Roche PhosSTOP phosphatase inhibitor cocktail, 5 mM sodium butyrate) was added, the solution was incubated on ice for 10 min, and the cells were pelleted by centrifugation at 300 × g for 3 min. To release nuclei, swollen cells were incubated in two volumes of Buffer E + inhibitors for 7 min and disrupted by Dounce homogenization using a B-type (tight) pestle. Nuclei were pelleted by centrifugation at 1500 × g for 3 min, and the supernatant (cytosolic extract) was transferred to a fresh tube. Nuclei were resuspended in 1 volume of Buffer E + inhibitors. An equal volume of Buffer N + inhibitors (20 mM HEPES, 10 mM potassium chloride, 1.5 mM MgCl_2_, 600 mM NaCl, 20% v/v glycerol, 1 mM DTT, 1× Roche EDTA-free protease inhibitor cocktail, 1× Roche PhosSTOP phosphatase inhibitor cocktail, 5 mM sodium butyrate) was added, and nuclei were salt-extracted on a rotator at 4 °C for 1 h. Extracted nuclei were centrifuged at maximum speed for 15 min at 4 °C to pellet chromatin, and the supernatant contained the nuclear extract.

### Affinity purification mass spectrometry (AP-MS) from nuclear extracts

For each pulldown reaction, 50 µL of uncoupled and Strep-Tactin^®^ Superflow^®^ high capacity resin (IBA-Lifesciences) were used. Beads were washed twice with 900 µL of PBS and once with 900 µL of wash buffer (20 mM HEPES pH 7.6, 0.3 M NaCl, 10% v/v glycerol, 0.05% v/v NP-40, 1× Roche EDTA-free protease inhibitor cocktail, 1× Roche PhosSTOP phosphatase inhibitor cocktail, 5 µM sodium butyrate). A total of 3.25 mg of nuclear extract (measured using the Bio-Rad protein assay) was used per reaction in pulldown buffer (20 mM HEPES pH 7.6, 0.3 M NaCl, 10% v/v glycerol, 0.5% v/v NP-40, 1× Roche EDTA-free protease inhibitor cocktail, 1× Roche PhosSTOP phosphatase inhibitor cocktail, 5 µM sodium butyrate). The sample was first added to uncoupled beads and incubated for 1 h at 4 °C with rotation to remove nonspecific binders. The sample was then centrifuged at maximum speed for 5 min, the supernatant was transferred to Strep-Tactin^®^ beads and incubated for 4 h at 4 °C with rotation, and the beads were washed three times for 5 min each with 900 µL of wash buffer. Protein complexes were eluted from the beads using two successive elutions by addition of 100 µL wash buffer supplemented with 4 mM desthiobiotin (IBA-Lifesciences) followed by shaking for 30 min at room temperature.

The eluates from the affinity pulldown reactions were buffer exchanged using Amicon Ultra 0.5 mL centrifugal filters with Ultracel 3K cutoff (Merck Millipore). The eluate (200 µL) was added and centrifuged at 14,000 × g for 30 min until the remaining volume was approximately 50 µL. Exchange buffer (50 mM Tris-HCl pH 8.0, 6 M guanidine-HCl, 3 mM β-mercaptoethanol) was added to a total volume of 500 µL, and the solution was centrifuged again. This process was repeated six times in total, and the final sample volume was 50 µL. The filter was then placed upside down in a protein lo-bind tube and centrifuged briefly to collect the sample. The sample was denatured at 95 °C for 20 min, and 250 µL of dilution buffer (50 mM ammonium bicarbonate, pH 7.8) was added to the sample to reduce the guanidine-HCl concentration. The solution was subjected to trypsin digestion, for which 12.5 µL of trypsin (20 µg/200 µL resuspension buffer) was added, and proteins were digested overnight at 37 °C. The next morning, the tubes were placed in an SC100 SpeedVac vacuum concentrator (Savant) on “high” for approximately 4 h.

Dried trypsin-digested peptides were reconstituted in 1 mL of 0.1% v/v heptafluorobutyric acid (pH 2.5), and C18 clean-up was performed using Sep-Pak cartridges (WAT054960) following the manufacturer’s instructions. Eluted peptides from each clean-up were evaporated to dryness in a SpeedVac and reconstituted in 20 µL of 0.1% v/v formic acid. Peptide samples were subjected to LC-MS/MS analysis on a Q Exactive Plus instrument (Thermo Fisher Scientific) interfaced with an UltiMate 3000 HPLC and autosampler system (Dionex). Peptides were separated using nano-LC, and the eluting peptides were ionized using positive ion mode nanoelectrospray ionization following the experimental procedures described previously.^44^ A survey scan range of *m/z* 350–1750 (automatic gain control (AGC) target = 1 × 10^6^) was recorded using an Orbitrap instrument (resolution = 70,000 at *m/z* 200) set to operate in DDA mode. Up to the 12 most abundant ions with charge states of > +2 were sequentially isolated and fragmented using higher-energy C-trap dissociation with the following parameters: normalized collision energy = 30, resolution = 17,500, maximum injection time = 125 ms, and AGC target = 1 × 10^5^. Dynamic exclusion was enabled (exclusion duration = 30 s).

### AP-MS data analysis

LC-MS/MS raw files were analyzed using MaxQuant version 1.6.10.^45^ Sequence database searches were performed using Andromeda,^46^ and the MaxLFQ algorithm^47^ was used to quantify proteins across samples. The following parameters were used. Precursor ion and peptide fragment mass tolerances were ±4.5 ppm and ±20 ppm, respectively. Carbamidomethyl (C) was included as a fixed modification, and oxidation (M) and N-terminal protein acetylation were included as variable modifications. Enzyme specificity was set at trypsin with up to two missed cleavages. The Swiss-Prot database (accessed March 2022, 566,996 sequence entries) was searched using only human sequences. The minimum peptide length was set as seven, the “match between runs” feature was enabled, and the MaxLFQ analyses were performed using default parameters with “fast LFQ” enabled. Protein and peptide false discovery rate thresholds were set at 1%. Datasets were further processed using Perseus^48^ to remove proteins within the MaxQuant contaminant database.^45^ For statistical analysis fold changes across sample groups were assessed using log transformed (base 2) MaxLFQ values, with significance determined using Student’s *t* tests.

### Chromatin preparation and affinity pulldown

Cells were detached with PBS supplemented with 125 mM EDTA. Cells were crosslinked in growth medium containing 1.2% v/v formaldehyde for 10 min, after which 125 mM glycine was added to stop the fixation process. Cells were washed three times with ice-cold PBS and resuspended in 2 mL of ice-cold swelling buffer (25 mM HEPES pH 7.6, 15 mM NaCl, 10 mM KCl, 2 mM MgCl_2_, 0.2% v/v NP-40, 1 mM EDTA, 0.5 mM EGTA, 0.5 mM DTT, 1× Roche EDTA-free protease inhibitor cocktail, 1× Roche PhosSTOP phosphatase inhibitor cocktail, 5 µM sodium butyrate) and incubated on ice for 10 min. This step was followed by addition of 2 mL of solution II (0.6 M sucrose, 120 mM KCl, 15 mM HEPES pH 7.6, 15 mM NaCl, 2 mM MgCl_2_, 0.2% v/v NP-40, 1 mM EDTA, 0.5 mM EGTA, 0.5 mM DTT, 1× Roche EDTA-free protease inhibitor cocktail, 1× Roche PhosSTOP phosphatase inhibitor cocktail, 5 µM sodium butyrate), and nuclei were released by Dounce homogenization using a B-type (tight) pestle. The cell suspension was overlaid onto 8 mL of buffer III (sucrose cushion: 1.2 M sucrose, 60 mM KCl, 15 mM HEPES pH 7.6, 15 mM NaCl, 2 mM MgCl_2_, 0.2% v/v NP-40, 1 mM EDTA, 0.5 mM EGTA, 0.5 mM DTT, 1× Roche EDTA-free protease inhibitor cocktail, 1× Roche PhosSTOP phosphatase inhibitor cocktail, 5 µM sodium butyrate) and centrifuged at 3900 × g for 38 min to pellet nuclei. The nuclei pellet was resuspended in 670 µL of sonication buffer (50 mM Tris-HCl pH 7.6, 1 mM EDTA, 0.5 mM EGTA, 1% w/v SDS, 0.1% w/v sodium deoxycholate, 1% v/v Triton-X-100, 1× Roche EDTA-free protease inhibitor cocktail, 1× Roche PhosSTOP phosphatase inhibitor cocktail, 5 µM sodium butyrate). The sample was sonicated in a Bioruptor on “high” with 30 s on/off cycles for a total of 60 min and then centrifuged at 10,000 × g for 15 min. The supernatant containing small chromatin fragments was transferred to fresh tubes and the concentrations measured by absorbance at 260 nm using a NanoDrop spectrophotometer.

For each pulldown reaction, 50 µL of uncoupled and Strep-Tactin^®^ Superflow^®^ high capacity resin (IBA-Lifesciences) were used. A total of 1.3 mg of chromatin was used per reaction in pulldown buffer (50 mM Tris-HCl pH 7.6, 0.1 M NaCl, 1% v/v NP-40, 0.5% w/v sodium deoxycholate, 0.1% w/v SDS, 1× Roche EDTA-free protease inhibitor cocktail, 1× Roche PhosSTOP phosphatase inhibitor cocktail, 5 µM sodium butyrate). The sample was incubated with uncoupled beads for 1 h at 4 °C with rotation to remove nonspecific binders and centrifuged at maximum speed for 5 min, and the supernatant was transferred to Strep-Tactin^®^ beads and incubated overnight at 4 °C with rotation. The sample was washed three times for 5 min each with 900 µL of wash buffer (100 mM Tris-HCl pH 7.6, 0.15 M NaCl, 1 mM EDTA, 1× Roche EDTA-free protease inhibitor cocktail, 1× Roche PhosSTOP phosphatase inhibitor cocktail, 5 µM sodium butyrate). Protein–DNA complexes and input chromatin were de-crosslinked and eluted from the beads by incubation in 95 µL of Tris-EDTA (TE) (0.1:10) with 1 µL of RNase (Roche) for 30 min at 37 °C, followed by addition of 2.5 µL 20% w/v SDS and 2.5 µL Proteinase K (20 mg/mL), and incubation at 65 °C for 10 h. To purify de-crosslinked chromatin, 100 µL of de-crosslinked DNA was added to 170 µL of AMPure beads (Beckman Coulter Life Sciences) equilibrated to room temperature. The mixture was incubated at room temperature for 15 min to bind DNA and placed over a magnet for 2 min. The supernatant was removed and the beads were washed twice with 350 µL of 80% v/v ethanol. The beads were dried at room temperature. DNA was eluted with 50 µL of TE (0.1:10) and quantified using the Qubit™ dsDNA HS Assay Kit (Invitrogen), as per the manufacturer’s protocol.

### ChIP-qPCR

ChIP-qPCR was performed using 1 µL of purified DNA for each reaction, as for RT-qPCR. The primers used for qPCR are listed in Table S3 (Supplementary material).

### DNA sequencing and analysis

DNA sequencing was performed by GENEWIZ (Suzhou, China). DNA was fragmented by acoustic disruption using an S200 Ultrasonicator (Covaris). Indexed libraries were prepared using a TruSeq DNA Nano kit (Illumina) according to the manufacturer’s protocol, and AMPure beads (Beckman Coulter Life Sciences) were used for clean-up and size selection. Libraries were sequenced on the Illumina HiSeq platform in a 150 base pair (bp) paired-end configuration at a depth of 330–400 million reads per lane. bcl2fastq (v2.17) was used for demultiplexing. Raw FASTQ sequencing data for each library were processed using FASTP version 0.19.5^49^ to remove potential adapter sequences, trim low quality bases, and perform quality control. The trimmed and quality-checked reads were then aligned to the unmasked human reference genome build GRCh38 (Ensembl annotation) using bowtie2 2.3.5^50^ with minor modifications to the default parameters (“–no-mixed” and “–no-discordant”) and allowing for a maximum insert size of 500 bp. The resulting SAM output was processed further using Samtools version 1.9^51^ and Picard Tools version 2.20.1. In a first step, alignments with a quality score <10 were removed, and duplicate reads were marked and removed using Picard tools “MarkDuplicates.” The sorted, quality-filtered, and deduplicated BAM files were than indexed using Samtools “index,” and insert size distributions estimated using Picard tools “CollectInsertSizeMetrics.”

Aligned ChIP-Seq and input data were inspected further using a set of quality control tools provided as part of the “deepTools” software package (version 3.2.0). In particular, pairwise Pearson correlations of read coverages were calculated and used to estimate the technical variability between replicates. Fingerprint plots were generated to estimate enrichment achieved in the ChIP libraries. deepTools was also used to create bigWig files for visualization in Integrative Genomics Viewer (IGV) (bamCoverage), and these bigWig files were used for calculating enrichment scores over input coverage (bigWigCompare), which were then visualized for selected genomic regions using computeMatrix and plotHeatmap/plotProfile.

Significantly enriched regions/peaks were identified using quality-filtered and deduplicated BAM files from each library by performing peak calling using MACS version 2.1.2.^52^ Input libraries were pooled as per MACS peak-calling best practice. Parameters for MACS were modified further from the default. The significance of the called peaks was determined by calculating the irreproducible discovery rate (IDR) in a second step; in this step, the q-value cutoff was increased to 0.99, which resulted in an almost complete list of all potentially enriched peaks/regions. The genome size parameter was set to “hs,” and summits were called. The called peaks for each replicate were then used as input for IDR (version 2.0.4.2), with “–use-best-multisummit-IDR” to account better for enrichment across potentially larger regions (1 kb+).

### Statistical analysis

Statistical analysis was performed using GraphPad Prism 9 (GraphPad Software Inc.). Differences between two groups were analyzed using the *t* test, and values of *p* < 0.05 were considered to be significant. All data are presented as mean ± standard deviation (SD).

## Supplementary Material

Supplemental Material

## Data Availability

The mass spectrometry proteomics data have been deposited to the ProteomeXchange Consortium via the PRIDE^53^ partner repository with the dataset identifier PXD033229. The ChIP-sequencing data are available in the ArrayExpress database (http://www.ebi.ac.uk/arrayexpress) under accession number E-MTAB-11617.
