# Supplementary material for: ANP32e Binds Histone H2A.Z in a Cell Cycle-Dependent Manner and Regulates Its Protein Stability in the Cytoplasm
Source: Mol Cell Biol. 2024 Mar 14;44(2):72–85. doi: 10.1080/10985549.2024.2319731 (PMC10950284; doi:10.1080/10985549.2024.2319731)
Supplement: Supplemental Material [file TMCB_A_2319731_SM2738.zip › tmcb-2023-0188-20240213191857/suppl_data/Table S1 S2.docx]

Table S1. Primer sequences for RT-qPCR

| Name | Sequence (5’-3’) |
| --- | --- |
| B-ACTIN_qPCR_1_F | CCAACCGCGAGAAGATGA |
| B-ACTIN_qPCR_1_R | TCCATCACGATGCCAGTG |
| GAPDH_qPCR_1_F | CATCAATGACCCCTTCATTG |
| GAPDH_qPCR_1_R | GATCTCGCTCCTGGAAGATG |
| ANP32e_qPCR_3_F | GGTATTGGGGAAGGCAGGAAA |
| ANP32e_qPCR_3_R | GGACTAACTCTGTCACCTCCTC |

Table S2. Primers sequences for ChIP-qPCR

| Name | Sequence (5’-3’) |
| --- | --- |
| TRPC_2_F | CAGCAGTGGGAACGACTCA |
| TRPC_2_R | CCATCATCGCGGCCCAT |
| CDKN2AIP_2_F | TTGGTCTTTAGGCCTGCGG |
| CDKN2AIP_2_R | GACAGGCAGGACTAGCGG |
| SOX14_2_F | GGGAGCCACGACTGGAAAC |
| SOX14_2_R | TGGGACCCAAGTATCGGAGT |
| ALCAM_2_F | CTTCAACCACCTGCTTTGCG |
| ALCAM_2_R | GGACTGGAAGGCGTGTGTAA |
| HERC3_2_F | CTGTGACAGTCGGAGTCCC |
| HERC3_2_R | CTGCAGCTCCGCCGTA |
| SMARCA2_2_F | CGGAGCCCGAGTTTAGGAAG |
| SMARCA2_2_R | AGGAGTGTGCTGGCTGAATC |
| CCDC71L_2_F | GTCCCAGTCCGCGTTGG |
| CCDC71L_2_R | GATATTCGCCCGCGTCCTC |
| PIEZO1_2_F | CTCTTCCTCCTTCTCCTTCGG |
| PIEZO1_2_R | CCCAGTGAGCCGAGCG |
| PRDM16_2_F | TTCTGGACTCAAGGAGGAGGAG |
| PRDM16_2_R | GAGACTTACTTTTGGCTAGCTTCCT |
| CDKN1A_2_F | CTTGGGCTGCCTGTTTTCAG |
| CDKN1A_2_R | GCTGGCAGATCACATACCCT |
