# Supplementary figures and images for: ANP32e Binds Histone H2A.Z in a Cell Cycle-Dependent Manner and Regulates Its Protein Stability in the Cytoplasm
Source: Mol Cell Biol. 2024 Mar 14;44(2):72–85. doi: 10.1080/10985549.2024.2319731 (PMC10950284; doi:10.1080/10985549.2024.2319731)

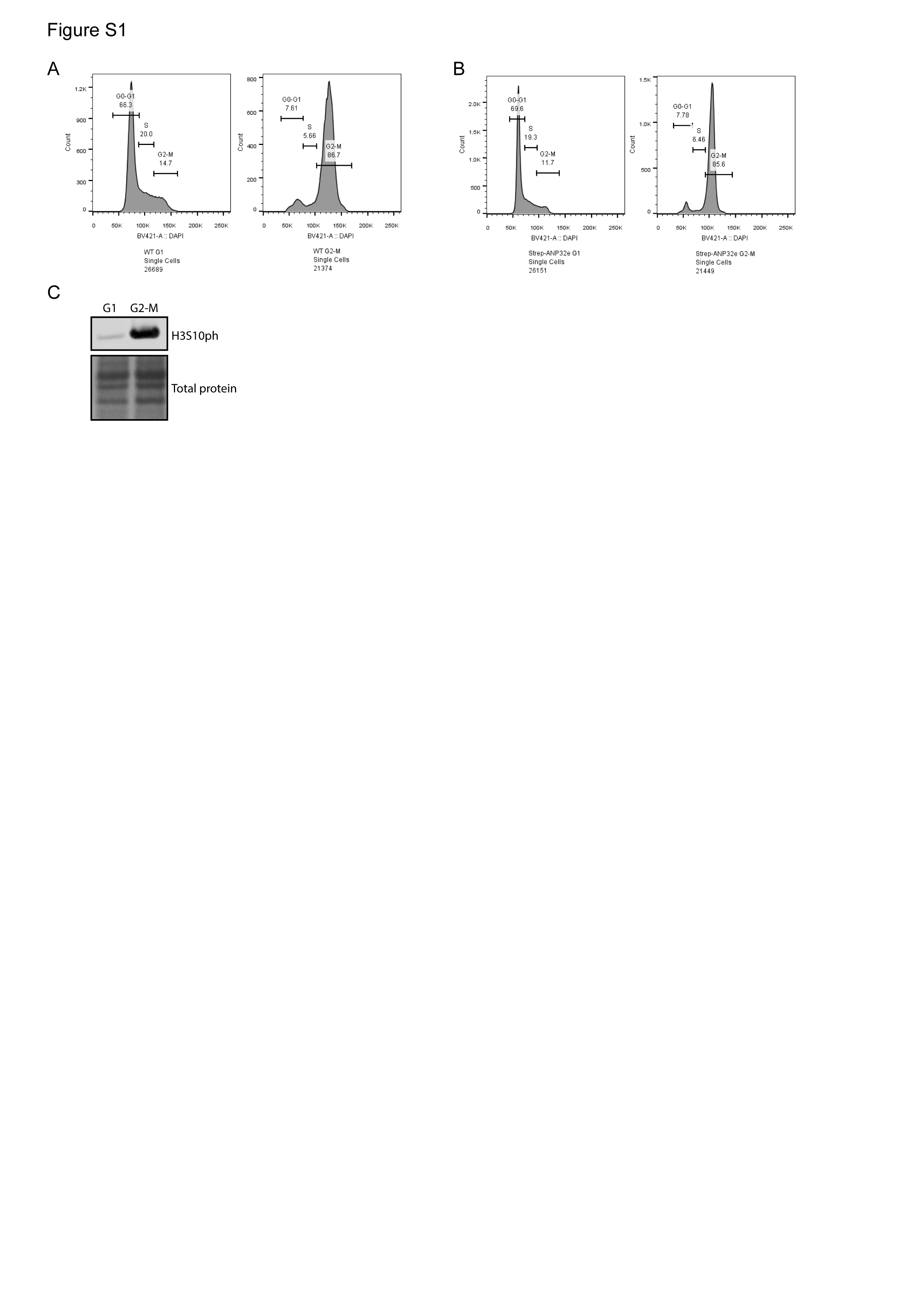

Supplement: Supplemental Material [file TMCB_A_2319731_SM2738.zip › tmcb-2023-0188-20240213191857/suppl_data/Figure S1.tif]

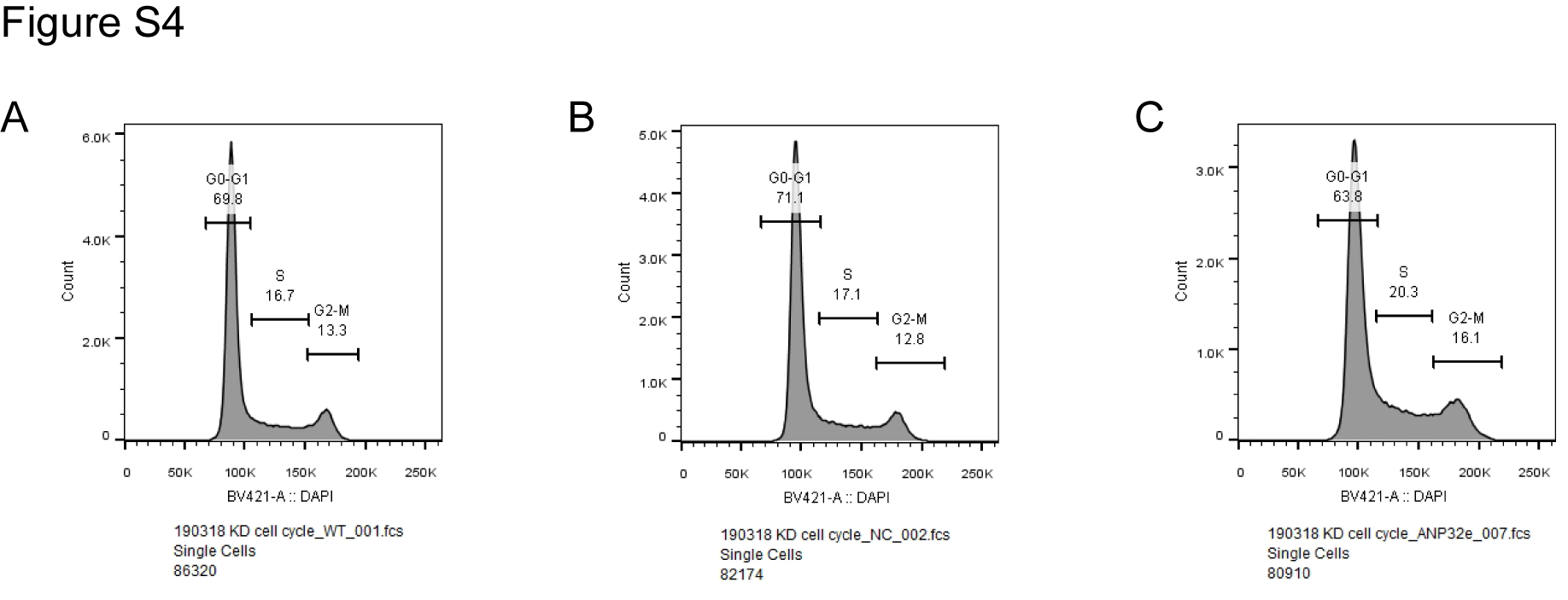

Supplement: Supplemental Material [file TMCB_A_2319731_SM2738.zip › tmcb-2023-0188-20240213191857/suppl_data/Figure S4.tif]

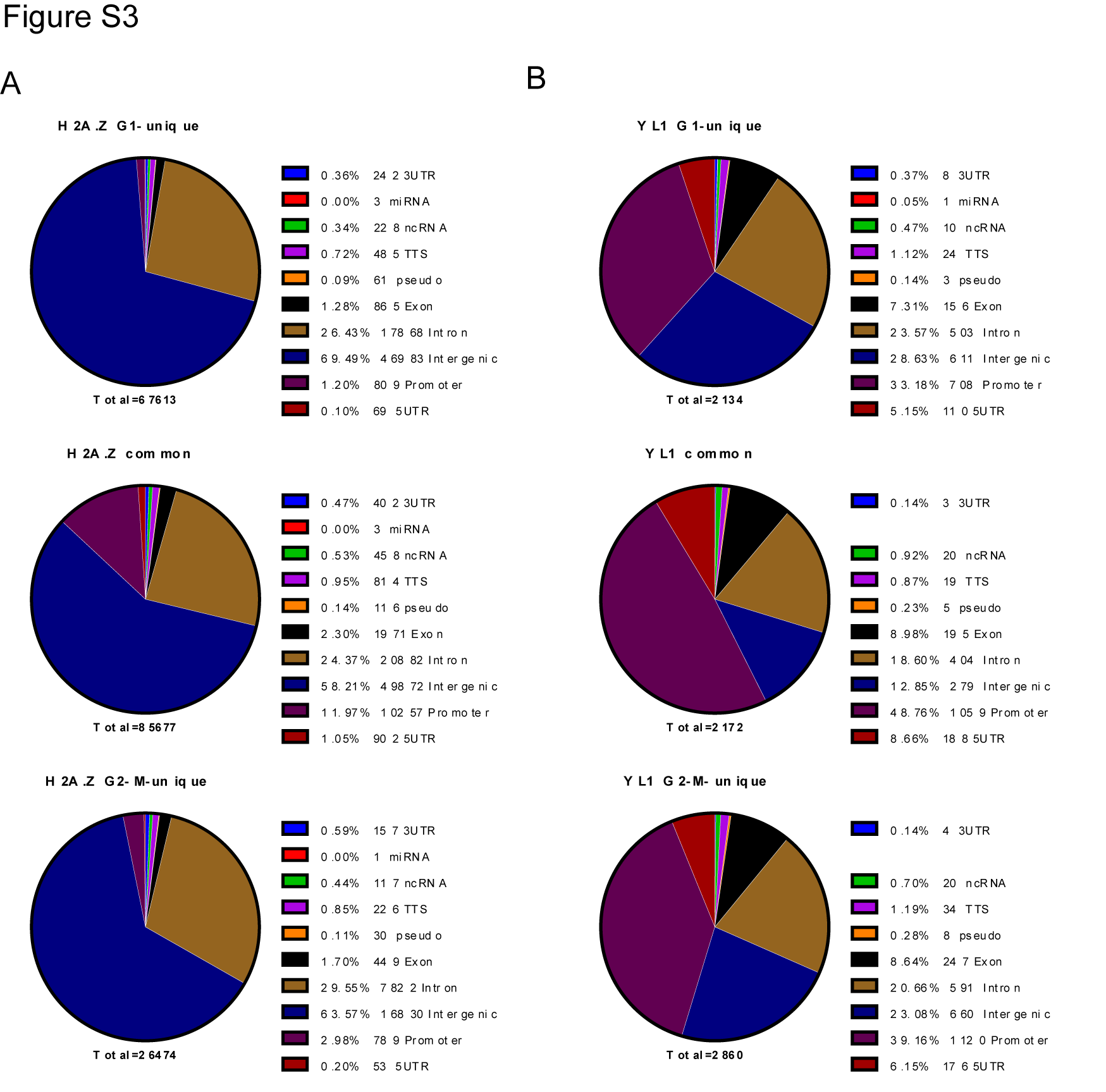

Supplement: Supplemental Material [file TMCB_A_2319731_SM2738.zip › tmcb-2023-0188-20240213191857/suppl_data/Figure S3.tif]

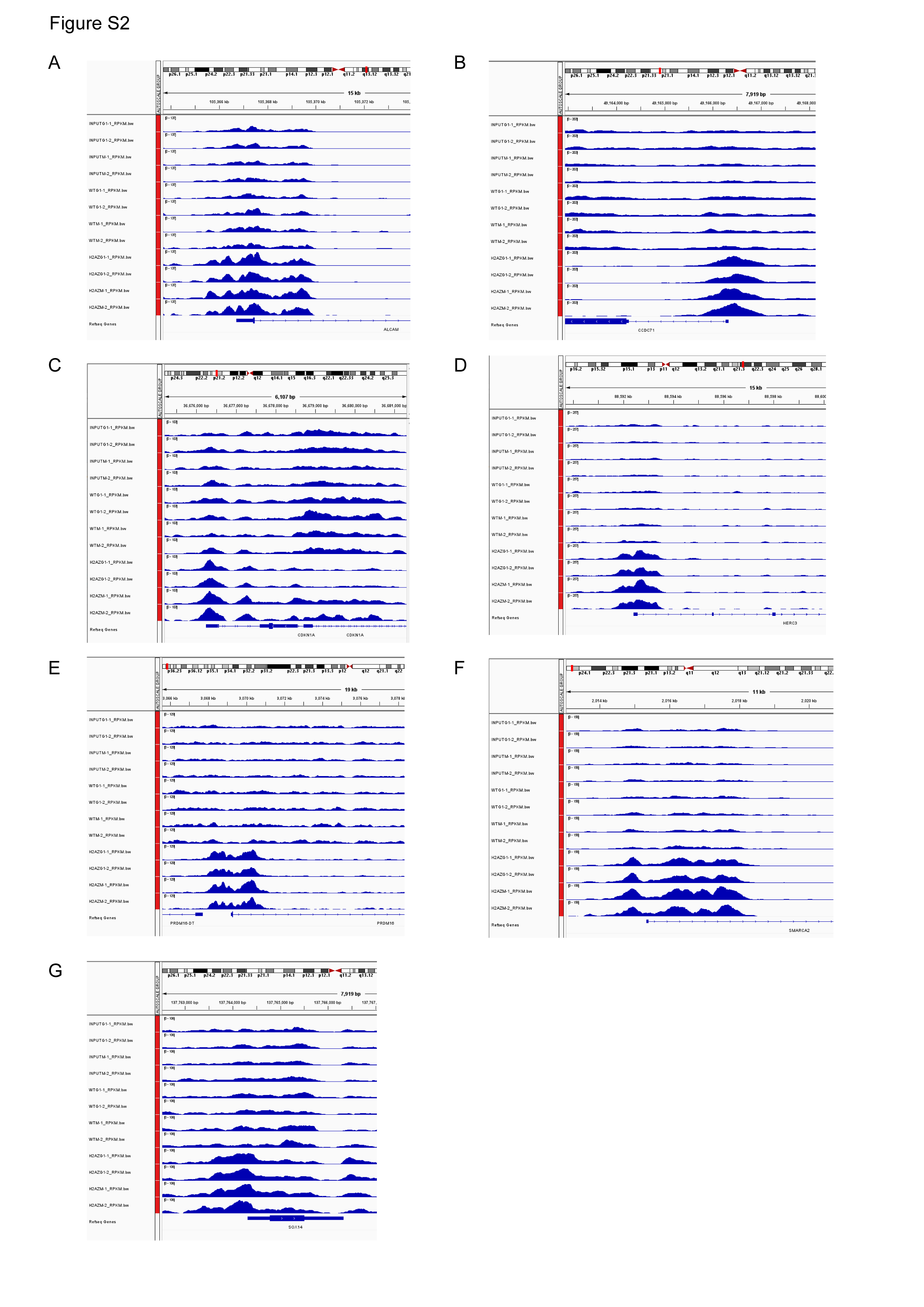

Supplement: Supplemental Material [file TMCB_A_2319731_SM2738.zip › tmcb-2023-0188-20240213191857/suppl_data/Figure S2.tif]
